# Exercise without dietary changes alleviates nonalcoholic fatty liver disease without weight loss benefits

**DOI:** 10.1186/s12944-018-0852-z

**Published:** 2018-09-01

**Authors:** Duck-Pil Ok, Kangeun Ko, Ju Yong Bae

**Affiliations:** Laboratory of Exercise Biochemistry, Department of Physical Education, College of Arts and Physical Education, Dong-A University, 37 Nakdong-daero 550 beon-gil, Hadan-dong, Saha-gu, Busan, 604-714 Republic of Korea

**Keywords:** NAFLD, Training, CB1 receptor, AMPK, FAS, CPT1

## Abstract

**Background:**

This study aimed to analyze the effect of exercise and/or dietary change on improvement of non-alcoholic fatty liver disease (NAFLD) in chronic high-fat diet (HFD)-induced obese mice.

**Methods:**

Forty male C57BL/6 (8 weeks old) mice were divided into normal diet (CO, *n* = 8) and high-fat diet (HF, *n* = 32) groups. The HF group was fed with 60% fat chow for 16 weeks to induce obesity. After the obesity induction period, the HF group was subdivided into HFD + sedentary (*n* = 8), HFD + training (HFT, *n* = 8), dietary change to normal-diet + sedentary (HFND, *n* = 8), and dietary change to normal-diet + training (HFNDT, *n* = 8) groups, and the mice in the training groups underwent treadmill training for 8 weeks, 5 times per week, 40 min per day.

**Results:**

A 24-week HFD induced increase of cannabinoid-1 receptor (CB1), fatty acid synthase (FAS), and AMP-activated protein kinase (AMPK) protein expressions (*p* < 0.05) and decrease of p-AMPK and carnitine palmitoyltransferase1 (CPT1) protein expressions (*P* < 0.05), resulting in increased liver fat accumulation. Treatment of exercise with dietary change and dietary change alone decreased CB1 and AMPK protein expressions with increased p-AMPK and CPT1 protein expressions (*P* < 0.05), leading to decreased body weight and liver fat (*P* < 0.05). The CB1 and FAS protein expressions in the HFT group were still higher than those in the CO group (*P* < 0.05), but the p-AMPK and CPT1 protein expressions were higher than those in the HF group (*P* < 0.05). Moreover, improved glucose tolerance and decreased liver fat were confirmed, although treatment of exercise alone had no effect on weight loss compared to pre-exercise.

**Conclusions:**

Even in the case of obesity induced by chronic HFD, exercise and/or dietary interventions have preventive and therapeutic effects on fat accumulation in the liver, resulting from upregulations of lipolytic factors. Therefore, the results of this study suggested that treatment of exercise alone without dietary change also leads to improvement of NAFLD and glucose tolerance without weight loss benefits.

## Background

Fatty liver disease is characterized by accumulation of lipids, mainly triglycerides, in hepatocytes in the absence of competing liver disease etiologies, which is an early stage of liver disease, such as alcoholic liver disease, non-alcoholic fatty liver disease (NAFLD), and hepatitis C [1]. Both pathological conditions, including central obesity, type 2 diabetes, dyslipidemia, hypertension, and lifestyle-related factors, such as physical inactivity and high-fat diets, are risk factors for the development of NAFLD [2]. The overall global prevalence of NAFLD was estimated to be 25%, and the prevalence of NAFLD in Asian populations is estimated to be 27% [3]. Because the prevalence of NAFLD is increasing worldwide and presents a public health burden, appropriate solutions are required for the alleviation of NAFLD.

Among the proposed mechanisms of NAFLD progression, lipogenic transcription factor activation [4] and impaired functions of lipolytic transcription factor [5] are directly related to the development of fatty liver. Cannabinoid-1 receptor (CB1), which responds to cannabinoids that increase fat intake, regulates the activity of these lipogenic and lipolytic transcription factors [6, 7], and the intake of ethanol and high-fat diet induce upregulation of CB1 activity through increased synthesis of endocannabinoids, 2-AG, and anandamide [1]. Because CB1 is also upregulated in obesity, CB1 is a potential therapeutic target for obesity and NAFLD [8].

The CB1 antagonist Rimonabant (Acomplia, Sanofi-Aventis) was initially developed for the treatment of obesity, and its effect was better than expected [9–11]. However, safety of Rimonabant was of concern because of the occurrence of central adverse effects, including nausea, anxiety, sleep disturbances, and depression, and clinical trials were eventually discontinued [12]. Although claims for drug development have been raised that CB1 antagonists should encourage ongoing efforts to develop peripherally restricted molecules that will prevent potential adverse central effects [8], there is no remarkable achievement to date.

Previous studies reported that a certain level of weight reduction is necessary to alleviate NAFLD [13, 14]. Exercise is one of the most effective ways to reduce body weight and fat accumulation by facilitating metabolic processes without any side effects. Dietary restriction or dietary conversion to low calories is also well known as an effective treatment for weight loss. Thus, regular exercise and dietary intervention have been recommended to alleviate NAFLD through weight reduction. A few studies examined the effects of exercise with dietary restriction on improvement of NAFLD, but the effect of the treatment of exercise alone that does not induce weight reduction is still unclear.

Therefore, the purpose of this study was to analyze the effect of exercise and/or dietary change on improvement of NAFLD in chronic high-fat diet (HFD) induced obese mice.

## Methods

### Animals and maintenance

Forty male C57BL/6 (8 weeks old) mice were used in this study. Four mice were housed per cage in the Dong-A University College of Medicine Animal Laboratory. The laboratory conditions were maintained constant: 55% relative humidity, 22 ± 2 °C, and a 12-h dark–light cycle. The animal experiments were approved by the Dong-A University Medical School Institutional Animal Care and Use Committee (DIACUC-approval-16-17), and all procedures were performed in accordance with the committee guidelines.

### Obesity induction

The animals were randomly divided into two groups to induce obesity: normal diet + sedentary group (ND, *n* = 8) and HFD + sedentary group (HF, *n* = 32). For 16 weeks, the HF group was fed with 60% fat chow (60% lipid, 20% carbohydrate, and 20% protein) to induce obesity, whereas the CO group was fed with a standard chow (6.3% lipid, 69.4% carbohydrate, and 24.3% protein). Body weight was measured weekly during the entire experimental period.

### Exercise and dietary intervention

After 16 weeks of obesity induction, the mice in the HF group were randomly subdivided into HF (*n* = 8), HFD + training (HFT, *n* = 8), dietary change to normal diet + sedentary (HFND, *n* = 8), and dietary change to normal diet + training (HFNDT, *n* = 8) groups. Animals in the HFT and HFNDT groups underwent exercise training on an animal treadmill five times per week for eight weeks. The exercise intensity was adjusted to 5 m/min for 5 min, 12 m/min for 30 min, and 5 m/min for 5 min, at 0% slope for the first four weeks of training. The intensity of exercise was increased to 5 m/min for 5 min, 14 m/min for 30 min, and 5 m/min for 5 min, also at 0% slope for the last four weeks of training [15].

### Glucose tolerance test

As previously described [16], an intraperitoneal glucose tolerance test (GTT) was performed after a 16-h overnight fast. Plasma glucose concentrations were measured in tail blood using a GlucoDr Blood Glucose Test Strip (Allmedicus, Anyang, South Korea) before and 30, 60, 90, and 120 min after intraperitoneally injecting a bolus of glucose (1 mg/g) for the GTT.

### Tissue sampling

Tissue sampling was conducted 48 h after the completion of the last exercise to prevent temporary training effects. Food was removed from the mouse cages 12 h before the mice were sacrificed. Liver tissues were excised after complete anesthesia (ethyl ether), and extracted samples were immediately weighed, frozen in liquid nitrogen, and stored at − 80 °C.

### Hematoxylin and eosin staining

Small pieces of liver tissue were fixed with formalin (10% neutral-buffer formalin) and embedded in paraffin. Five-micrometer sections were cut and stained using hematoxylin and eosin (H&E). Digital images of the slides were captured with an Aperio ScanScope (Aperio, USA).

### Extraction of liver triglycerides

Liver samples were weighted (50 mg), and 200 μl of ethanolic KOH (2 parts ethanol: 1 part 30% KOH) was added to each sample and incubated overnight at 55 °C. Subsequently, it was mixed with 50% ethanol into each tube to bring the volume to 0.5 ml and centrifuged for 5 min at 13,000 rpm. The supernatant was transferred into new tubes, and 50% ethanol was added again into each tube to bring the volume to 0.6 ml. After vortexing, 200 μl was moved to a new tube, and 215 μl of 1 M MgCl_2_ was added, incubated for 10 min on ice, and centrifuged for 5 min at 13,000 rpm. The supernatant was used to measure liver TG using ASAN set Triglyceride-S Reagent (Asan Pharmaceutical, Seoul, South Korea) by the enzymatic colorimetric method. Liver lysates and standards were added into the microplate and incubated at 37 °C for 10 min. Absorbance values were measured at 550 nm.

### Western blotting

As previously described [15], the liver tissues were lysed in 200 μl radioimmunoprecipitation assay (RIPA) buffer to extract protein from the samples. The tissue was homogenized and centrifuged for 30 min at 14,000 rpm. The protein concentration of the supernatant was measured using the BCA protein assay kit (PIERCE, USA). Samples of equal protein content were resolved by SDS-polyacrylamide gel electrophoresis on a 10 or 12% gel and transferred to a membrane. The membrane was blocked with 5% skim milk in phosphate-buffered saline (PBS), and subsequently incubated at 4 °C overnight with primary antibodies (1:1000 dilution) against CB1 (sc-293419), fatty acid synthase (FAS, sc-74540), carnitine palmitoyltransferase1 (CPT1, sc-393070) (all from Santa Cruz Biotechnology, USA), AMP-activated protein kinase (AMPK, #2532, Cell Signaling Technology, USA), and phosphor-AMPK (p-AMPK, #2531, Cell Signaling Technology). The membrane was incubated with goat anti-mouse or anti-rabbit IgG conjugated secondary antibody for 1 h at room temperature. The signal was developed with an ECL solution (Amersham Pharmacia Biotech, USA) and visualized with ImageQuantTM LAS-4000 system (GE Healthcare, Sweden).

### Statistical analysis

All statistical analyses were performed with Statistical Package for Social Sciences (version 22.0); values were presented as means±SE. To compare groups, we performed analysis of variance, using the least-square difference post hoc test to validate significant differences. A significance level of *p* = 0.05 was used as a threshold for statistical significance.

## Results

### Chronic HFD induced obesity, glucose tolerance, and fat accumulation in liver

**Fig. 1:**
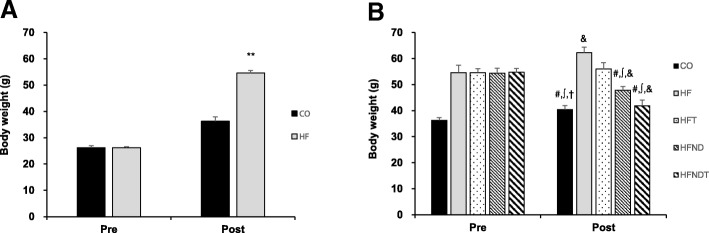
Changes in body weight after intervention. Changes in body weight after 16-week HFD (**a**) and 8-week training and/or dietary change (**b**). Data are expressed as mean ± SE. CO, normal-diet group; HF, high-fat diet group; HFT, high-fat diet + training group; HFND, dietary change to a normal diet group; HFNDT, dietary change to a normal diet + training group. ** versus CO group, *p* < 0.001; ^#^ versus HF group, *p* < 0.05; ^∫^ versus HFT group, *p* < 0.05; ^†^ versus HFND group, *p* < 0.05; ^&^ versus before, *p* < 0.05

**Fig. 2:**
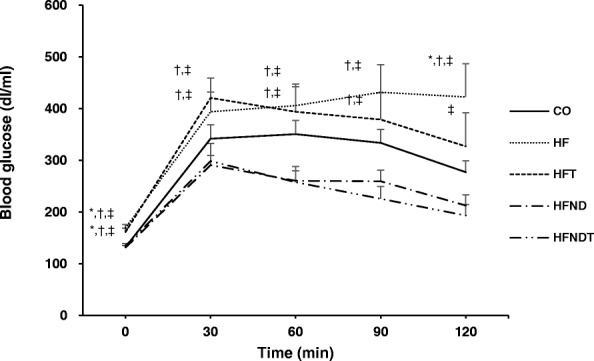
Glucose tolerance test after 8-week training and/or dietary change. Changes in blood glucose after 8-week intervention. Data are expressed as mean ± SE. CO, normal-diet group; HF, high-fat diet group; HFT, high-fat diet + training group; HFND, dietary change to a normal diet group; HFNDT, dietary change to a normal diet + training group. * versus CO group, *p* < 0.05; ^†^ versus HFND group, *p* < 0.05; ^‡^ versus HFNDT group, *p* < 0.05

**Fig. 3:**
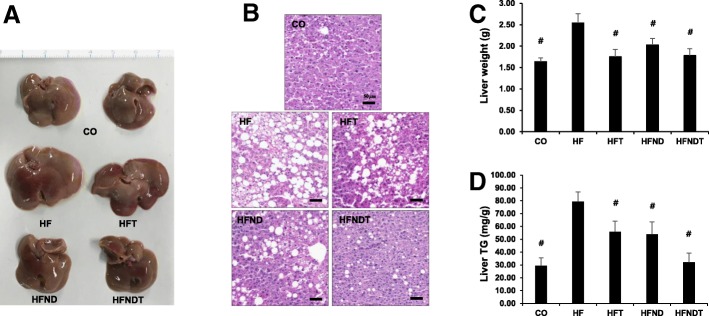
Changes of liver after 8-week training and/or dietary change. Changes in liver size (**a**), lipid droplet (**b**), liver weight (**c**), and liver triglyceride (**d**) after treadmill training. Data are expressed as mean ± SE. CO, normal-diet group; HF, high-fat diet group; HFT, high-fat diet + training group; HFND, dietary change to a normal diet + sedentary group; HFNDT, dietary change to a normal diet + training group. ^#^ versus HF group, *p* < 0.05. Scale bar = 50 μm

Body weight in the HF group was significantly higher than that in the CO group after 16 weeks of HFD (*p* < 0.001); thus, we determined that obesity was induced in the HF group (Fig. 1a). Body weight in the HF group was significantly higher than that in all other groups after eight weeks of exercise and/or dietary intervention (*p* < 0.05) (Fig. 1b), and the blood glucose level in the HF group tended to increase over time (Fig. 2). Furthermore, chronic HFD increased liver weight (Fig. 3c) and liver TG (Fig. 3d) (*p* < 0.05).

### Both exercise and/or dietary intervention alleviates body weight gain, glucose tolerance, and fat accumulation in liver

Eight weeks of regular exercise and/or dietary conversion showed improvement in obesity caused by chronic HFD. The body weight in the HFT, HFND, and HFNDT groups was significantly lower than that in the HF group (*p* < 0.05) (Fig. 1b). Dietary change groups achieved weight loss effects regardless of whether exercise was conducted. The body weight in the HFT group did not decrease, but the prophylactic effect of weight gain was observed.

The blood glucose levels in the HF and HFT groups were significantly higher than those in the HFND and HFNDT groups during most periods of GTT (*p* < 0.05). However, blood glucose in the HFT group tended to decrease over time, suggesting that exercise without dietary conversion is somewhat effective in blood glucose control (Fig. 2). Therefore, dietary conversion is considered to be essential for gaining the maximum benefit from blood glucose control.

Eight weeks of exercise and/or dietary intervention induced significant decrease of liver weight (Fig. 3c) and liver TG (Fig. 3d) in both training and/or dietary change groups (*p* < 0.05).

### Both exercise and/or dietary intervention alleviates fatty liver by regulation of protein expressions related fat accumulation in liver

**Fig. 4:**
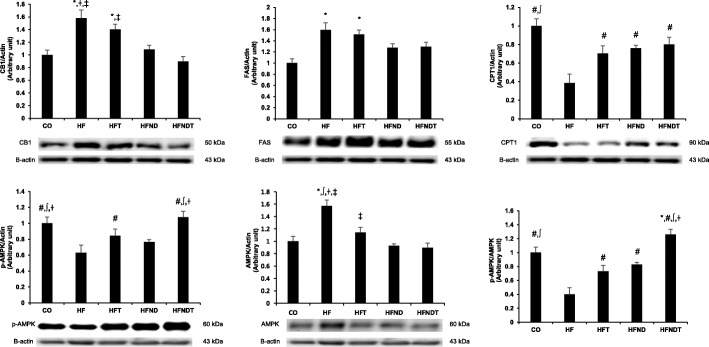
Protein expressions in the liver after 8-week training and/or dietary change. Protein expressions in the liver after 8-week intervention. Data are expressed as mean ± SE. CO, normal-diet group; HF, high-fat diet group; HFT, high-fat diet + training group; HFND, dietary change to a normal diet group; HFNDT, dietary change to a normal diet + training group. * versus CO group, *p* < 0.05; ^#^ versus HF group, *p* < 0.05; ^∫^ versus HFT group, *p* < 0.05; ^†^ versus HFND group, *p* < 0.05; ^‡^ versus HFNDT group, *p* < 0.05

Protein expression related to fat accumulation in the liver after eight weeks of training are presented in Fig. 4. The protein expression of CB1, FAS, and AMPK was significantly higher, and CPT1 and p-AMPK were significantly lower in the HF group than those in the CO group (*p* < 0.05). Although the protein expression of CB1 and FAS in the HFT group was not significantly different from that in the HF group, the protein expression of CPT1, p-AMPK, and AMPK in the HFT group was significantly higher than those in the HF group (*p* < 0.05). In the protein expression of the HFND group, CB1 and AMPK were significantly lower, and CPT1 was significantly higher than those in the HF group (*p* < 0.05). In the protein expression of the HFNDT group, CB1 and AMPK were significantly lower, and CPT1 and p-AMPK were significantly higher than those in the HF group (*p* < 0.05).

## Discussion

In this study, we confirmed that CB1 and FAS protein expressions were increased in liver tissue of chronic HFD mice, and AMPK and CPT1 protein expressions were decreased. After combination of regular exercise and dietary change intervention, however, fat synthesis decreased and beta oxidation of fatty acids increased. Moreover, treatment of exercise alone also induced improvement of liver weight, liver TG with beta oxidation of fatty acids increased without benefits of weight loss.

The overall prevalence of NAFLD worldwide and in Asian populations was estimated to be > 25% [3] and has also sharply increased over the past several decades in Korean populations [17]. The progression of NAFLD is usually slow over and more a decade, and it is asymptomatic in most cases [18], and 10–20% of NAFLD patients eventually progress to not only non-alcoholic steatohepatitis, but also multi-organ systemic diseases [17].

Excess fat accumulation in liver could result from (a) increased de novo fatty acid synthesis, (b) increased transport of fatty acids from the other organs to the liver, (c) decreased fatty acid oxidation, and (d) decreased transport of triglycerides from the liver to other organs [1]. In this respect, exceed lipid in the body due to HFD could directly or indirectly promotes fat accumulation in the liver. Evidence showing that the increase of lipogenic factors and decrease of lipolytic factors induced fat accumulation in the liver was well known, through both human [19, 20] and animal studies [21, 22]. In this study, as expected, chronic HFD increased lipogenic factor, such as FAS with decreased lipolytic factors, such as AMPK and CPT1, which induced increased fat accumulation, size, and liver weight.

Emerging evidence suggests that cannabinoids play an important role in the regulation of fatty liver [1, 8, 23]. The main endocannabinoids (endogenous cannabinoids) discovered are anandamide and 2-arachidonoylglycerol, and its receptors have been identified are CB1 and cannabinoid receptor 2 [1]. A previous study reported that an HFD increases hepatic levels of anandamide, CB1 protein level, and basal rates of fatty acid synthesis, and the latter is reduced by CB1 blockade [24]. The mechanism underlying these effects is that hepatocytes express CB1, stimulation that induces the increase of de novo fatty acid synthesis resulting from the expressions of SREBP-1c and its target enzymes [24, 25]. Furthermore, researchers have reported that CB1 knockout mice were totally resistant to HFD-induced obesity and steatosis [26–28]. These studies clearly support the important role of CB1 receptors in HFD-induced fatty liver and obesity. In this study, 16 weeks of HFD induced increase of FAS protein expression, which acts as a lipogenic factor, and decrease of lipolytic factors, such as AMPK and CPT1 through upregulation of CB1, resulting in accumulation of liver fat. However, although CB1 protein expression was upregulated by chronic HFD, treatment of exercise alone without dietary change upregulated lipolytic factors and inhibited liver fat accumulation.

The high prevalence of NAFLD had provided a challenge for many researchers, resulting in the conducting of studies on treatment strategies for NAFLD [29–32]. Because NAFLD patients are usually obese and have insulin resistance, lifestyle modification and pharmacologic therapies to regulate the body weight and related target enzymes and hormones have been proposed as treatment for NAFLD. Body weight loss of at least 3% to 5% is required to reduce liver steatosis [13, 14], for this reason, lifestyle modification for weight loss, such as exercise and/or dietary change intervention, is recommended as the first intervention of NAFLD. Kenneally et al. (2017) conducted a study of efficacy of dietary and physical activity intervention in NAFLD, suggesting that combination of moderate dietary restriction and 30–60 min of moderate–intensity exercise was effective in reducing NAFLD activity. In trials evaluating dietary modification or exercise intervention alone, dietary intervention alone was enough to reduce body weight, whereas exercise intervention alone was not [32]. Nevertheless, exercise trials lead to an improvement in NAFLD by significantly reducing one or more markers of NAFLD without weight loss [33–35]. This study also showed that combined treatment and dietary change treatment alone were effective in improving fatty liver through inhibition of CB1. Particularly, it is noteworthy that improvement of glucose tolerance and decreased liver TG were observed, although exercise intervention alone had no effect on weight loss compared with pre-exercise. In our previous study, regular exercise alone did not reduce body weight but improved insulin resistance [36]. In fact, observing the effects of regular exercise on weight loss during the growing period when the body weight gradually increases is difficult. Therefore, for improving NAFLD, associating not only body weight reduction but also changes in body composition, such as body mass, muscle mass, and fat mass may be necessary.

## Conclusion

Even in the case of obesity induced by chronic HFD, exercise and/or dietary intervention had preventive and therapeutic effects on fat accumulation in the liver, resulting from upregulations of lipolytic factors. Therefore, the results of this study suggested that treatment of exercise alone without dietary change also led to improvement of NAFLD and glucose tolerance without benefits of weight loss.

## Abbreviations

NAFLD: Non-alcoholic fatty liver disease
HFD: High-fat diet
CB1: Cannabinoid 1 receptor
AMPK: AMP-activated protein kinase
FAS: Fatty acid synthase
CPT1: Carnitine palmitoyltransferase1
